# Unveiling Self-Organization and Emergent Phenomena in Urban Transportation Systems via Multilayer Network Analysis

**DOI:** 10.3390/e27111169

**Published:** 2025-11-19

**Authors:** Hongqing Bao, Xia Luo, Xuan Li, Yiyang Zhao

**Affiliations:** 1 National Engineering Laboratory of Integrated Transportation Big Data Application Technology, Chengdu 611756, China; baiba@my.swjtu.edu.cn (H.B.); lxwjk@my.swjtu.edu.cn (X.L.); zhaoyy@my.swjtu.edu.cn (Y.Z.); 2 School of Transportation and Logistics, Southwest Jiaotong University, Chengdu 611756, China

**Keywords:** self-organization, emergence, simplicity and complexity, temporal urban mobility network, multimodal transport nodes

## Abstract

In the absence of system-wide planning and coordination, emerging mobility services have been integrated into urban transportation systems as independent network layers. Meanwhile, their interactions with traditional public transit give rise to complex self-organizing patterns in population mobility, manifested as coopetitive dynamics. To systematically analyze this phenomenon, this study constructs a four-layer temporal network—consisting of ride-hailing, metro, combined, and potential layers—based on a vectorized multilayer network model and inter-layer mapping relationships. An analytical framework is then developed using node strength, cosine similarity, and rich-club coefficients, along with two newly proposed indicators: the intermodal index and the node importance coefficient. The results reveal, for the first time, a spontaneously emergent intermodal phenomenon between ride-hailing and metro networks, manifested through both cross-day modal substitution and intra-day intermodal chains. The analysis further demonstrates that when sufficiently large and homogeneous demand cohorts are present, the phenomena can emerge even on non-working days. Based on the characteristics of this phenomenon, a method has been developed to identify intermodal nodes across different transport networks. Furthermore, the study uncovers a time-varying multicentric hierarchical structure within the metro network, characterized by small-scale core rich nodes and larger-scale secondary rich-node clusters. Overall, this study provides novel insights into the formation, coordination, and optimization of intermodal urban transport networks.

## 1. Introduction

A complex system is generally defined as a system composed of two or more interacting components [1]. Intermodal transportation can be regarded as an instance of such a complex system, characterized by the use of two or more transportation modes to complete a single journey [2,3]. This approach effectively alleviates urban traffic burden and congestion issues by optimizing the capacity and connectivity between different transportation modes [4,5,6]. Ride-hailing users and public transit users typically demonstrate a high degree of group overlap [7]. In recent years, numerous researchers have conducted in-depth analyses of intermodal solutions that integrate ride-hailing with public transportation using modeling and simulation approaches [8,9,10,11,12]. These studies have indicated that well-designed intermodal systems can not only significantly enhance the modal share of public transportation but also yield substantial socio-economic benefits.

However, due to the lack of unified planning and coordination across different transportation networks [13], the ride-hailing and public transportation networks in real-world systems exhibit a complex coopetition relationship [14,15,16,17], which is considered an adaptive processes that constitute self-organized intermodal networks [18]. Previous studies based on big data-driven statistical analysis and structured questionnaire-based survey analysis have shown that ride-hailing services can both extend the coverage of public transit and attract potential riders [19,20,21] while also potentially diverting public transit ridership due to superior user experience or limited metro accessibility [22,23,24]. Although these studies have revealed the phenomenon of coopetition, the statistical analyses—typically based on regression coefficients or causal inference—are inherently limited in capturing the intrinsic interaction mechanisms of transportation networks at both the topological and dynamic flow levels. Survey-based studies, on the other hand, are constrained by biases in self-reported data and issues of sample representativeness. Consequently, empirical research has yet to provide a comprehensive understanding of the temporal heterogeneity in coopetition interactions and their driving mechanisms.

Network science offers a systematic framework for uncovering the underlying mechanisms of urban transportation systems by adopting a multilayer network perspective, encompassing aspects such as topological structure, functional coupling, and dynamic evolution [25,26]. As a result, vector-based multilayer network models constructed from multimodal transportation systems have been widely adopted [27]. Within this framework, researchers typically divide the system into the Physical Infrastructure Layer and the Flow Layer that captures the movement dynamics on top of the infrastructure [28]. Some studies further differentiate layers based on types of transport modes [29]. By employing complex network metrics—such as degree distribution, betweenness centrality, clustering coefficient, and network robustness—these studies systematically analyze the structural characteristics and vulnerabilities of urban transportation networks [30,31,32,33,34,35,36].

However, existing multilayer network studies on intermodal self-organizing phenomena primarily focus on modes such as bike-sharing and public transportation, which more readily facilitate the spontaneous formation of intermodal travel chains [37,38,39], while analysis of major urban travel modes—such as ride-hailing and public transportation—remains insufficient. In this study, the author constructed a four-layer temporal network of ride-hailing, metro, combined, and potential networks to analyze the interaction characteristics of real-world networks at the dynamic flow level. This approach elucidated the mechanisms underlying modal substitution and the emergence of self-organized intermodal networks. Furthermore, the author identified key intermodal nodes based on the rich-club phenomenon in four-layer networks. The contributions of this study are as follows.

(1) The author found that, even without unified planning and coordination, self-organization can emerge between ride-hailing and metro networks when supported by a sizable and homogeneous demand cohort. Such coopetition manifests as mode substitution across different days and as intermodal chains within a single day.

(2) The author constructed a potential multimodal network by generating intermodal travel chains in direct competition with actual ride-hailing trajectories, thereby quantifying the latent demand for cross-network coordinated travel in real-world systems.

(3) Building on the in strength and out strength of nodes across four layers, the author proposed a multilayer network framework for analyzing coopetition interactions, integrating cosine similarity, the rich-club coefficient, and the intermodal index and node importance coefficient defined in this study.

(4) In the analysis of the rich-club phenomenon, the author found that transportation networks exhibit a hierarchical structure consisting of the small-scale core rich nodes and the larger-scale secondary rich nodes. This hierarchical structure is particularly significant in the metro network.

## 2. Data Preparation and Multilayer Network

One concept commonly associated with complex systems is emergence, which refers to self-organization arising from the local interactions among system components [40]. A typical manifestation of self-organization in transportation systems is traffic congestion. In contrast, studies on self-organized intermodal phenomena remain limited due to the difficulty of acquiring suitable data. This study selected Chengdu as a case study to examine urban population mobility between metro and ride-hailing networks. Although the analysis is confined to a single city, the proposed framework can be applied to any city with analogous data.

### 2.1. Data Description and Preprocessing

Chengdu, the capital of Sichuan Province, covers an area of 14,335 km^2^ and had a resident population of 19.81 million as of 2018. This study utilizes Didi ride-hailing GPS trajectories and Chengdu Metro passenger flow records collected between 1 November and 30 November 2018. The observation period includes 22 working days and 8 non-working days, with no additional public holidays. According to the operational schedule of the Chengdu Metro, only data within the 15 h window from 06:00 to 21:00 were considered valid for analysis.

Table 1 presents the structure of the Didi ride-hailing trajectory data used in this study, which includes geographic coordinates, timestamps, and vehicle status identifier codes. Code 0 indicates that the vehicle is in an unoccupied state (without passengers, either cruising or stationary waiting for passengers); code 1 denotes that the vehicle is in an occupied state (with passengers on board, executing mobility services). The data is sampled every 3 s, with coordinate accuracy at the meter level, which satisfies the data requirements of this investigation. It is important to note that due to the continuous 24 h operation of ride-hailing services, the trajectory data for the 30th day may be statistically incomplete. The day was excluded from further analysis.

Due to the lack of specific passenger information in the ride-hailing trajectory data, this study infers the origin–destination (OD) passenger flow of ride-hailing services based on the following:

(1) Identifying a single ride: a change in the vehicle status identifier codes of the same vehicle (0 → 1 or 1 → 0) is marked as the start (O) and end (D) of an independent trip event.
(2) Standardization of OD trip units: all passengers within a single ride share the same origin and destination and are considered as a single OD trip unit, where each ride corresponds to one standardized OD trip unit.

This method may overlook OD diversity in carpooling scenarios with multiple passengers in the same vehicle during the same time period. However, in the absence of detailed passenger records, this assumption represents a reasonable simplification scheme that balances data availability against analytical feasibility.

Table 2 presents the information about Chengdu Metro. As of November 2018, the city had 6 operational metro lines with 136 stations.

Table 3 presents the structure of Chengdu Metro passenger flow data. Each record contains the following fields: Card ID (de-identified information), Card Type, Entry Station ID, Entry Station Name, Entry Time, Exit Station ID, Exit Station Name, and Exit Time.

### 2.2. Temporal Urban Mobility Network

Temporal network theory enables the analysis of passenger flow dynamics at urban or regional scales—capabilities that static network theory lacks [41]. Recognizing the limitation of static representations, it becomes crucial to extend network analysis to time-varying structures [42]. Based on previous research, this study established a directed weighted mobility network using time-varying passenger flows as weights [43]. However, existing analyses of temporal urban transportation networks often neglect the underlying physical topology, hindering the examination of inter-modal relationships within the same spatial context. To address this, this study introduced mapping relations between networks to establish the potential intermodal network.

#### 2.2.1. Temporal Chengdu Metro Network (CM)

**Figure 1:**
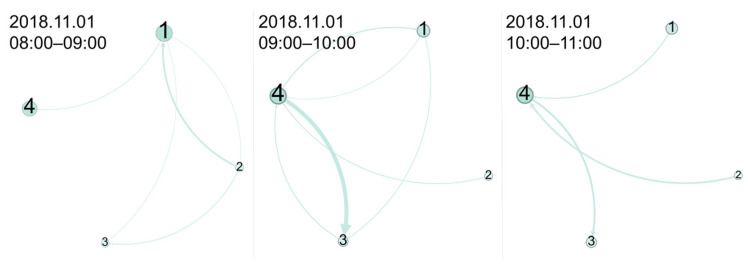
Hourly OD diagram between metro stations: 1 is Chengdu Academy of Governance station, 2 is Fuqing Road station, 3 is Xinglong Lake station, and 4 is Kuanzhaixiangzi Alleys station.

Based on the metro stations’ connectivity properties and passenger flows, this study established the temporal Chengdu Metro network, which is represented in the form of a directed weighted graph. Using the software Gephi 0.10.1, Figure 1 visualizes three hourly strength changes for selected metro stations.

(1) $$G_{t}^{CM}=\left(V^{CM},E^{CM},W^{CM}(t)\right)$$

(2) $$W^{CM}(t)=\left[\begin{array}{ccc} 0 & \ldots & w_{1n}^{CM}(t)\\ \vdots & \ddots & \vdots \\ w_{n1}^{CM}(t) & \ldots & 0 \end{array}\right]$$

where *V^CM^* denotes the set of *n* metro stations and *E^CM^* denotes the set of connectivity edges between metro stations (based on OD). *W^CM^* (*t*) denotes the directed weighted matrix used to include the strength *w^CM^* (*t*) between stations during different time periods *t* (one-hour interval). The diagonal values of *W^CM^* (*t*) are consistently 0, indicating that no OD passenger flows exist within the station. The hourly variations in strength distribution reveal different passenger migration patterns within the network.

#### 2.2.2. Temporal Ride-Hailing Network (RH)

According to previous studies, the temporal ride-hailing network can be determined by partitioning the road network into units [44]. To construct the CM&RH network and potential intermodal network, the partitioning of the road network must adhere to the following constraints:

(1) Single-station constraint: no road network unit can contain more than one metro station;
(2) Service radius constraint: the side length of each unit should match the service radius (walking accessibility area) of the metro station.

**Figure 2:**
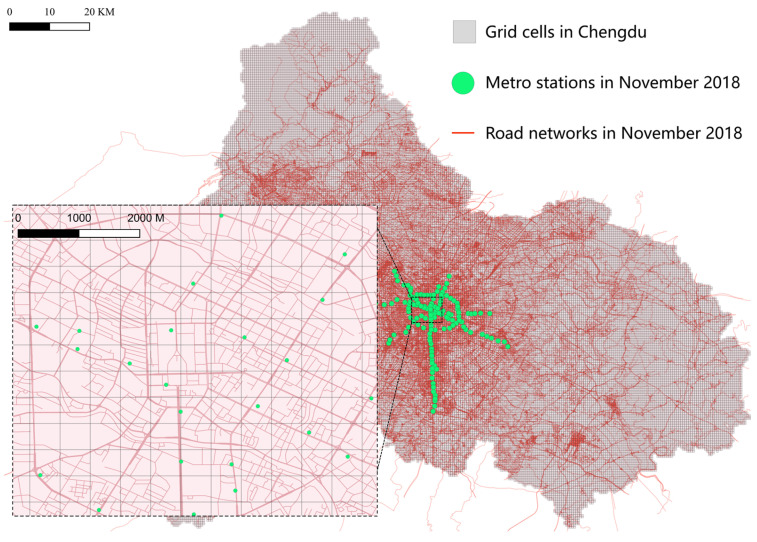
Road network and metro stations in Chengdu in 2018. Each grid cell has a side length of 500 m.

Figure 2 shows all road network units, with an edge length set at 500 m, totaling 58,897 units. Based on the connectivity properties between road network units and the preprocessed ride-hailing OD passenger flows, the author established the temporal ride-hailing network.

(3) $$G_{t}^{RH}=\left(V^{RH},E^{RH},W^{RH}(t)\right)$$

(4) $$W^{RH}(t)=\left[\begin{array}{ccc} w_{11}^{RH}(t) & \ldots & w_{1q}^{RH}(t)\\ \vdots & \ddots & \vdots \\ w_{q1}^{RH}(t) & \ldots & w_{qq}^{RH}(t) \end{array}\right]$$

where *V^RH^* denotes the set of *q* road network nodes and *E^RH^* denotes the set of connectivity edges between road network nodes (based on OD). *W^RH^*(*t*) includes the hourly ride-hailing strength *w^RH^*(*t*). Unlike the metro network, the diagonal weights of *W^RH^*(*t*) have numerical values, indicating that OD passenger flows exist within road network nodes.

#### 2.2.3. CM&RH Network (O)

The CM&RH network refers to the temporal metro network superimposed onto the temporal ride-hailing network. Due to the single-station constraint, each metro station corresponds uniquely to a single road network node. The strength in the CM&RH network is defined as the sum of the strength from both networks, with no transfer flows occurring between the two modes.

(5) $$G_{t}^{O}=\left(V^{O},E^{O},W^{O}(t)\right)$$

(6) $$W^{O}(t)=\left[\begin{array}{ccc} w_{11}^{O}(t) & \ldots & w_{1q}^{O}(t)\\ \vdots & \ddots & \vdots \\ w_{q1}^{O}(t) & \ldots & w_{qq}^{O}(t) \end{array}\right]$$

where *V^O^* denotes the set of *q* road network nodes and *E^O^* denotes the set of connectivity edges between road network nodes (based on OD). *W^O^*(*t*) includes the hourly strength *w^O^*(*t*), with diagonal weights having numerical values.

### 2.3. Mapping Between CM and RH

To construct the potential intermodal network, it is necessary to establish a bidirectional and uniquely mapped topological relationship between metro stations and road network units. However, some metro nodes are located near the boundaries of road network units, which may lead to inaccuracies in direct mapping. To address this issue, this study constructed the mapping relationship based on the spatial positioning of metro nodes within road network units and set the control group to assess the impact of mapping errors on the resulting indicators.

#### 2.3.1. Network Mapping

**Figure 3:**
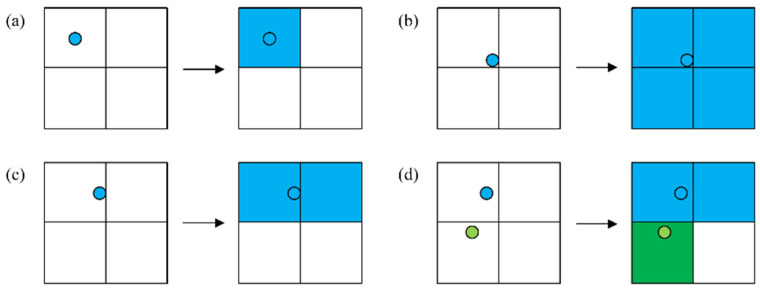
Network mapping rules. The circles represent metro stations, and the squares represent road network units. Each square has a side length of 500 m. Blue and green indicate different metro stations and their respective service areas. (**a**) Metro station is situated in the central region; (**b**) Metro station is located in the corner area; (**c**) Metro station is located in the side area; (**d**) Special cases.

Considering a 500 m radius as the mapping range for metro stations, this study set a threshold distance of 50 m to refine the mapping results. Specifically, if a metro station is located within 50 m of the boundary of its corresponding road network unit, a detailed mapping judgment is required. Figure 3 illustrates the differentiated mapping rules between metro network nodes and road network units.

(1) The central area mapping rule means that when a metro station is situated in the central region (greater than 50 m from the boundary), the station establishes bidirectional topological associations exclusively with its located unit (Figure 3a).
(2) The boundary area mapping rule is when a metro station is situated in the boundary region (less than 50 m from the boundary). Figure 3c shows the mapping rule if it is located in the side area, and Figure 3b shows the mapping rule if it is located in the corner area.

**Figure 4:**
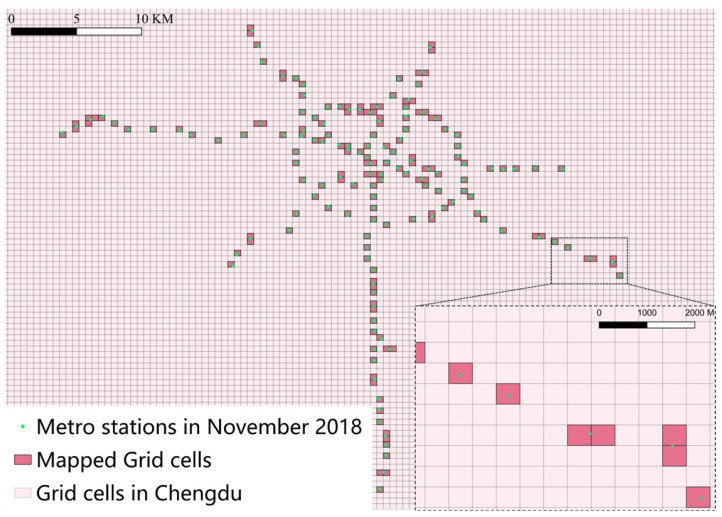
Mapped grid cells in Chengdu. Each grid cell has a side length of 500 m.

Furthermore, special cases exist among Chengdu’s metro stations. Figure 3d illustrates the positions and mapping relationships between Tonghuimen Station and Kuanzhaixiangzi Alley Station. For other similar cases, the study also redefined the mapping method. Figure 4 illustrates the mapping results, having 169 mapped road network units.

#### 2.3.2. Network Mapping Control Group

**Figure 5:**
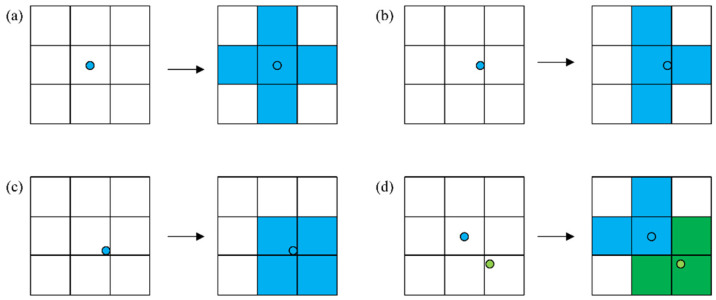
Fully saturated mapping rules for the control group. The circles represent metro stations, and the squares represent road network units. Each square has a side length of 500 m. Blue and green indicate different metro stations and their respective service areas. (**a**) Metro station is situated in the central region; (**b**) Metro station is located in the side area; (**c**) Metro station is located in the corner area; (**d**) Special cases.

In the control group, the critical distance is set to 200 m, meaning that all road network units within 800 m are included in the mapping range of each metro station. This corresponds to a coverage radius of 800 m and is defined as the fully saturated mapping group, which serves to examine how different mapping approaches may affect specific metric outcomes. Figure 5 illustrates the saturated mapping rules between metro nodes and road network units.

(1) The central area mapping rule is when a metro station is situated in the central region (greater than 200 m from the boundary); Figure 5a shows the mapping rule.
(2) The boundary area mapping rule is when a metro station is situated in the boundary region (less than 200 m from the boundary). Figure 5b shows the mapping rule if it is located in the side area, and Figure 5c shows the mapping rule if it is located in the corner area.

**Figure 6:**
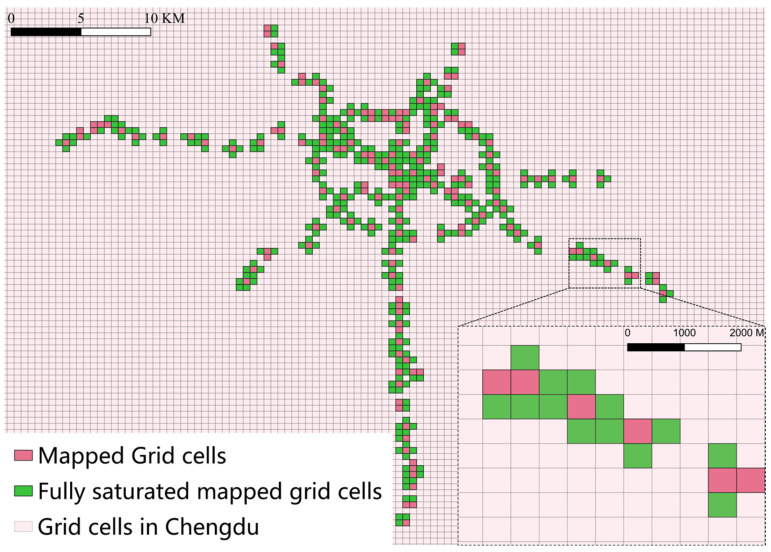
Fully saturated mapped grid cells. Each grid cell has a side length of 500 m.

In addition, special cases are handled as illustrated in Figure 5d. Figure 6 illustrates the saturated mapping outcome, having 544 mapped road network units.

### 2.4. Potential Intermodal Network (PI)

This study identifies the distribution of potential rich nodes associated with transfer passenger flows through the rich-club phenomenon within a real network. However, due to the independent operational characteristics of current transportation modes, the potential demand for cross-network has not been considered. The author proposes the concept of a Potential Intermodal Network by constructing intermodal travel chains to quantify such potential cross-network intermodal relationships.

The construction rules for intermodal travel chains are as follows: in the ride-hailing network, passengers whose routes pass through mapped units are assumed to transfer to the metro, forming the “RH-CM-RH” intermodal travel chain. The potential intermodal network is composed of the metro transfer links within these intermodal travel chains, with its network structure containing only metro stations as nodes, as formally expressed below.

(7) $$G_{t}^{PI}=\left(V^{PI},E^{PI},W^{PI}(t)\right)$$

(8) $$W^{PI}(t)=\left[\begin{array}{ccc} 0 & \ldots & w_{1q}^{PI}(t)\\ \vdots & \ddots & \vdots \\ w_{q1}^{PI}(t) & \ldots & 0 \end{array}\right]$$

**Figure 7:**
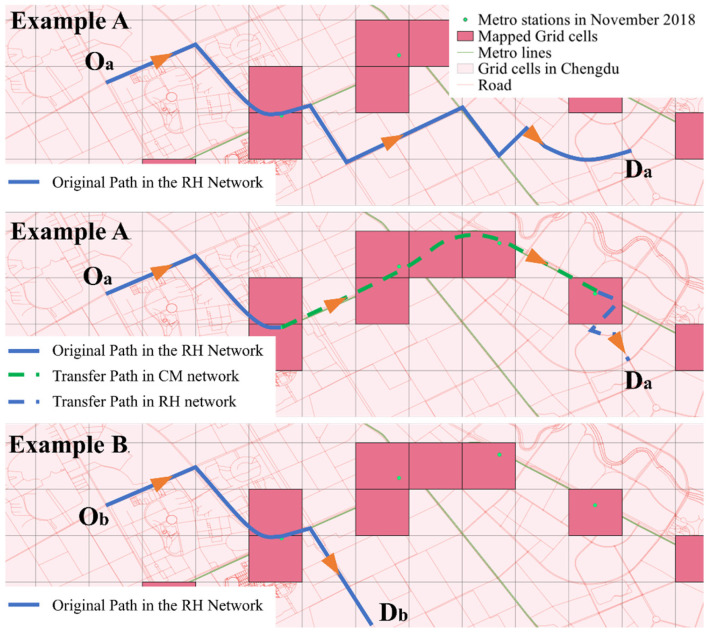
Examples from original to transfer path: Example A forms a transfer chain; Example B does not.

The construction of intermodal travel chains must satisfy the following constraints: identify the metro station closest to the passenger’s destination based on the shortest path algorithm, which must not be the same as the metro station corresponding to the mapped unit; if they are identical, no intermodal travel chain is generated. Figure 7 shows two cases when converting a ride-hailing route into an “RH-CM-RH” intermodal travel chain. The construction rules and constraints ensure that the intermodal travel chains establish a direct competition relationship with the real ride-hailing routes, thereby supporting the feasibility of route substitution.

## 3. Research Methods

### 3.1. Uncover Self-Organized Intermodality

In the study of single-layer networks, numerous classical metrics have been developed to reveal structural properties of networks, such as node similarity, rumor centrality, and eigenvector centrality [42]. As the research paradigm extends from single-layer networks to multilayer networks represented by multiplex networks, many studies have employed measures such as common neighbor or the Adamic–Adar index [45] to quantify the similarity between nodes within the same network. However, for multilayer networks with dynamic time-varying structures and lacking explicit inter-layer connections, modeling cross-layer associations and conducting similarity analysis remain relatively underexplored, and research in this area is still in its developmental stage.

The essence of node similarity lies in cosine similarity, a widely used metric for quantifying the similarity between two non-zero vectors [46,47]. In passenger flow matrices constructed based on time-varying flow weights, cosine similarity is often applied to measure the similarity between multiplex nodes. However, existing studies typically focus only on nodes shared across layers, resulting in a limited scope of similarity measurement [48]. To overcome this limitation, the present study introduces network mapping relations to extend cosine similarity to dynamically structured multilayer networks, allowing a more comprehensive characterization of cross-layer node associations and addressing gaps in previous research on temporal network dynamics and inter-layer dependencies.

Relying solely on a cosine similarity framework to analyze network phenomena may provide a limited perspective. Drawing inspiration from the construction of the Point of Interest Density Index [49], this study proposes the intermodal index, which, in conjunction with cosine similarity, constitutes a comprehensive similarity analysis framework for systematically investigating self-organization phenomena in intermodal networks.

#### 3.1.1. Strength Distribution

According to Yifan Zhang et al. [41], the strength *s_i_* of node *v_i_* refers to the sum of the weights *w_i_* of the connected edge set *E_i_* in the time period *t* ∈ ℝ*^H^*. The strength can be divided into the in strength *s^in^*(*t*)and out strength *s^out^*(*t*), as shown in Equations (9) and (10). The total strength *s*(*t*) is shown in Equation (11), and the total strength matrix *S*(*t*) is shown in Equation (12).

(9) $$s_{i}^{in}(t)=\sum_{j\neq i}w_{ji}(t)$$

(10) $$s_{i}^{out}(t)=\sum_{j\neq i}w_{ij}(t)$$

(11) $$s_{i}(t)=s_{i}^{in}(t)+s_{i}^{out}(t)$$

(12) $$S(t)=\left[\begin{array}{ccc} s_{11}(t) & \ldots & s_{1n}(t)\\ \vdots & \ddots & \vdots \\ s_{n1}(t) & \ldots & s_{nn}(t) \end{array}\right]$$

**Figure 8:**
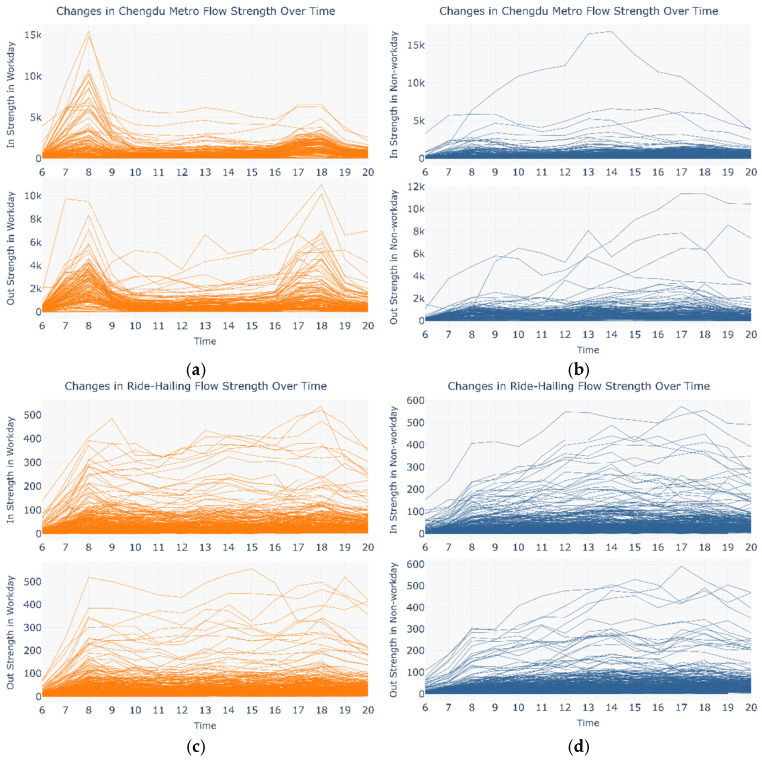
Changes in the in and out strength for CM and RH over time. (**a**) Distribution of in and out strength for CM on workdays; (**b**) distribution of in and out strength for CM on weekends; (**c**) distribution of in and out strength for RH on workdays; (**d**) distribution of in and out strength for RH on weekends.

Figure 8 shows the distribution of the in strength and out strength for CM and RH on workdays and weekends.

#### 3.1.2. Cosine Similarity

To reveal passenger flow similarities between different dates, this study employs cosine similarity to quantify directional consistency of feature vectors. Therefore, the total strength matrices *S* ∈ ℝ*^D^*^×*H*×*N*^ of three networks need to be transformed into feature vectors suitable for cosine similarity calculation. Specifically, by unfolding the hour dimension *H* and station dimension *N*, the three-dimensional feature matrix (Equation (13)) is flattened into a two-dimensional feature vector (Equation (14)).

(13) $$F_{d}=\left\{s_{d,h,n}\right\}\in {\mathbb{R}}^{D\times H\times N}$$

(14) $$x_{d}=\mathrm{vec}(F_{d})\in {\mathbb{R}}^{D\times \left(H\times N\right)}$$

where *s_d,h,n_* denotes the total strength at the *n*-th station during the *h*-th hour on the *d*-th day, *D* represents 30 days, *H* refers to the 15 h time window from 06:00 to 21:00, and *N* refers to either 136 metro stations or 58,897 road network units. Subsequently, a cosine similarity feature matrix is constructed (Equation (15)), followed by normalization processing (Equation (16)).

(15) $$X=\left[\begin{array}{c} x_{1}^{T}\\ x_{2}^{T}\\ \vdots \\ x_{D}^{T} \end{array}\right]\in {\mathbb{R}}^{D\times \left(H\times N\right)}$$

(16) $${\tilde{x}}_{d}=\left(x_{d}-\mu \right)/\sigma$$

Thus, a cosine similarity matrix *CS* ∈ ℝ*^D^*^×*D*^ is obtained. The closer the cosine similarity value *CS_ik_* (Equation (17)) is to 1, the more similar the passenger flow distribution between the two dates. Conversely, as the cosine similarity approaches −1, it indicates greater disparities in passenger flow distributions between the two dates.

(17) $$CS_{ik}=\frac{{\tilde{x}}_{i}\cdot {\tilde{x}}_{k}}{\left\|{\tilde{x}}_{i}\right\|\left\|{\tilde{x}}_{k}\right\|}$$

#### 3.1.3. Intermodal Index

In the intermodal transportation network, ride-hailing supports both first-mile and last-mile travel scenarios. The author clarifies the correspondence between in/out strength and two travel scenarios: in strength corresponds to the last mile and out strength corresponds to the first mile. The intermodal index quantifies the relative density of per mapping strength for ride-hailing passenger flow under these two scenarios. Abnormal fluctuations in the index may indicate distinct behavioral patterns. For instance, a significantly higher index during commuting hours compared to leisure periods suggests the emergence of a self-organized, commute-oriented intermodal network.

(18) $$II^{in}(t)=\frac{\left(\sum_{i=1}^{L}s_{i}^{in}(t)\right)\times \left(\sum_{i=1}^{Q}1(s_{i}^{in}(t)\neq 0)\right)}{\left(\sum_{i=1}^{Q}s_{i}^{in}(t)\right)\times \left(\sum_{i=1}^{L}1(s_{i}^{in}(t)\neq 0)\right)}$$

(19) $$II^{out}(t)=\frac{\left(\sum_{i=1}^{L}s_{i}^{out}(t)\right)\times \left(\sum_{i=1}^{Q}1(s_{i}^{out}(t)\neq 0)\right)}{\left(\sum_{i=1}^{Q}s_{i}^{out}(t)\right)\times \left(\sum_{i=1}^{L}1(s_{i}^{out}(t)\neq 0)\right)}$$

where time period is *t* ∈ ℝ*^H^*, *L* denotes all mapping units in the road network unit, and *Q* denotes all road network unit. *II^in^*(*t*) indicates the intermodal index for last-mile travel tasks and *II^out^*(*t*) indicates the intermodal index for first-mile travel tasks.

### 3.2. Identify the Key Intermodal Node

Based on the similarity analysis framework, this study revealed self-organization phenomena within networks. Building on the characteristics of these phenomena, this study further proposed a method for identifying key intermodal nodes in multilayer networks. Previous studies have predominantly relied on the rich-club algorithm to detect high-degree nodes in static network topologies; for dynamic networks with passenger flows, the rich-club algorithm could also identify key nodes based on flow strength [48,49,50,51,52]. However, due to the lack of explicit cross-layer connections, this approach could not be directly applied to multilayer networks. To address this limitation, this study constructed a potential intermodal network, introducing potential edges as cross-layer links, thereby enabling the identification of key nodes in temporally evolving multilayer networks. Furthermore, since the rich-club algorithm does not quantify the relative importance of key nodes, this study proposed the node importance coefficient to rank and evaluate these nodes quantitatively. Finally, the self-organization phenomena are validated through a significance analysis of line-level origin–destination flows.

#### 3.2.1. Rich-Club Coefficient

In weighted networks, node richness can be determined according to node strength. The global rich-club coefficient derived from node richness reflects the ratio between the actual connection strength among rich nodes and the theoretical maximum connection strength. According to Opsahl et al. [53], the global rich-club coefficient in a directed weighted network is extended as follows:

(20) $$\phi^{w}(r)=\frac{\sum_{x,y\in E}w_{xy}}{\sum_{k=1}^{\left|E\right|}w_{k}}$$

where parameter *r* defines the threshold of node richness, filtering rich nodes with strengt *s > r*. The set *E* denotes the edges existing exclusively among these rich nodes, *|E|* is the number of these edges, and *w_xy_* represents the weight of these edges. *w_k_* denotes the *k*-th largest weight when all edge weights in the network are arranged in descending order. A global rich-club coefficient closer to 1 at smaller *k* values indicates a more pronounced “rich-club” phenomenon.

To mitigate the influence of underlying network structures—such as degree and weight distributions—on the results, Colizza et al. [54] introduced a randomized null model and proposed the computation of a normalized rich-club coefficient.

(21) $$\rho (r)=\frac{\phi^{w}(r)}{\phi^{null}(r)}$$

The normalized rich-club coefficient *ρ*(*r*) reflects the ratio between the rich-club coefficient of the actual network *ϕ^w^*(*r*) and that of the randomized null model coefficient *ϕ^null^*(*r*). When *ρ* > 1, a positive rich-club phenomenon exists in the network.

(22) $$\rho_{k}^{in}(t)=\frac{\phi^{w}(s_{k}^{in}(t))}{\phi^{null}(s_{k}^{in}(t))}$$

(23) $$\rho_{k}^{out}(t)=\frac{\phi^{w}(s_{k}^{out}(t))}{\phi^{null}(s_{k}^{out}(t))}$$

The richness parameter *r* is defined in two ways: in strength *s^in^*(*t*) and out strength *s^out^*(*t*). This study adopts a dynamic threshold progression method to systematically identify a rich-node subset within the network. Specifically, the author ranks all network nodes by strength in descending order and sequentially selects the top *k* nodes (*k* = 1:*N*) as local richness thresholds. The richness parameter *r_k_*(*t*) in this method is defined as *s_k_*(*t*).

Subsequently, a set of normalized rich-club coefficients {*ρ_k_*(*t*)} is generated based on these local thresholds (as shown in Equations (22) and (23)), and the optimal rich-node clusters for both in strength and out strength are determined. Finally, the subset of rich nodes *V^O^*(*t*) with valid mapping relationships is selected, as shown in Equations (24) and (25), where the time period *t* is further categorized into commuting and leisure periods.

(24) $$V_{in}^{O}(t)=V_{\max(\rho )}^{CM,in}(t)\cap_{Map}V_{\max(\rho )}^{RH,in}(t)$$

(25) $$V_{out}^{O}(t)=V_{\max(\rho )}^{CM,out}(t)\cap_{Map}V_{\max(\rho )}^{RH,out}(t)$$

#### 3.2.2. Potential Intermodal Node

Although the “RH-CM-RH” intermodal mode has not yet been widely adopted in current operations, a potential intermodal network based on this model can be constructed through network reconstruction and simulation. Following a similar methodology as for real networks, the set of rich nodes *V^PI^*(*t*) within the potential intermodal network was calculated. A metro station is only identified as a potential intermodal node if it is recognized as a rich node in both real networks and the potential intermodal network.

(26) $$V_{in}(t)=V_{in}^{O}(t)\cap_{Map}V_{in}^{PI}(t)$$

(27) $$V_{out}(t)=V_{out}^{O}(t)\cap_{Map}V_{out}^{PI}(t)$$

#### 3.2.3. Node Importance Coefficient

This study introduces an Importance Coefficient (*IC* ∈ [0, 1]) to rank potential intermodal nodes across commuting and leisure periods. *IC* is defined as the relative frequency with which a node is identified as a potential intermodal node. Values of *IC* near 1 denote high cross-network intermodal potential, whereas values near 0 indicate limited intermodal engagement. Based on these definitions, *IC* is computed separately for four distinct cases (see Equations (28) and (29)), with the time interval set |*T*| partitioned into commuting and leisure periods as follows:

(1) *IC* of nodes serving first-mile tasks during commuting periods;
(2) *IC* of nodes serving first-mile tasks during leisure periods;
(3) *IC* of nodes serving last-mile tasks during commuting periods;
(4) *IC* of nodes serving last-mile tasks during leisure periods.

(28) $$IC_{in}^{T}(v)=\frac{\sum_{t\in T}1(v\in V_{in}(t))}{\left|T\right|}$$

(29) $$IC_{out}^{T}(v)=\frac{\sum_{t\in T}1(v\in V_{out}(t))}{\left|T\right|}$$

## 4. Results and Discussion

### 4.1. Emergence of Modal Substitution and the Intermodal Network

At the individual level, mode substitution is manifested when a user alternates between metro and ride-hailing services on different days (e.g., metro on Monday, ride-hailing on Tuesday). If such substitutability is pronounced at the citywide level, the constructed CM&RH network demonstrates practical relevance. Given the sparse ride-hailing data and the disparity in data volume between the two networks (1.8 × 10^4^ vs. 3.5 × 10^9^), the author employs the direction-sensitive, scale-invariant cosine similarity measure to evaluate mode substitutability.

**Figure 9:**
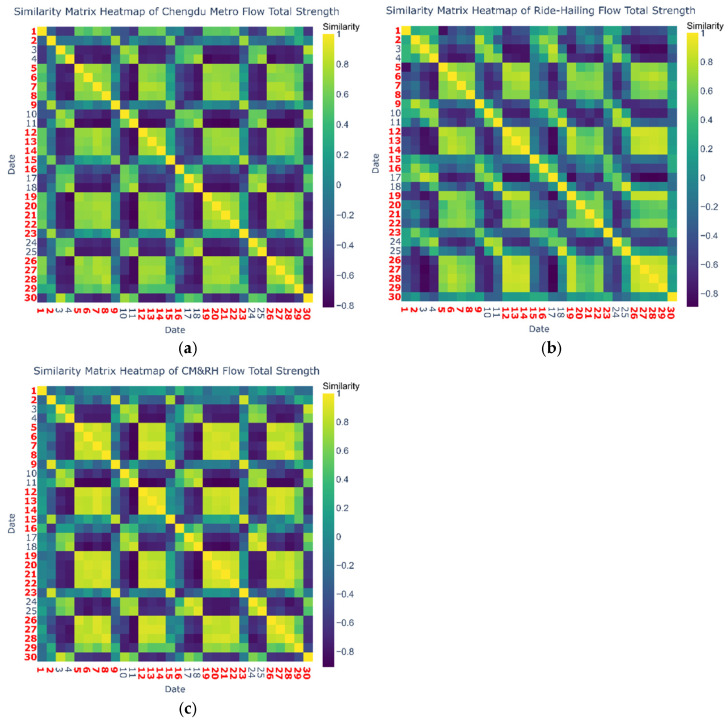
Cosine similarity matrix heatmap of three networks, the red numerals indicate workdays. (**a**) Cosine similarity matrix heatmap of CM; (**b**) cosine similarity matrix heatmap of RH; (**c**) cosine similarity matrix heatmap of CM&RH.

Figure 9 presents a heatmap of the cosine similarity matrix across the three networks, using an hourly node-by-node comparison (*H* × *N*). The red-labeled date numbers denote workdays; other dates denote weekends. Overall, the three networks exhibit highly similar daytime flow patterns on workdays. Moreover, as shown in Figure 8, the ride-hailing network displays significant differences in strength distribution between workdays and weekends, indicating that the commuting characteristics of ride-hailing users on workdays are relatively similar to those of metro users.

Simultaneously, the three networks show similar weekly passenger flow trends, but there exist substantial differences between workdays and weekends. Notably, Friday’s flow pattern differs significantly from other workdays. Based on these observations, travel days were grouped into three categories, Monday to Thursday, Friday, and the weekend, and then the cosine similarity within each category was recalculated (see Table 4).

**Figure 10:**
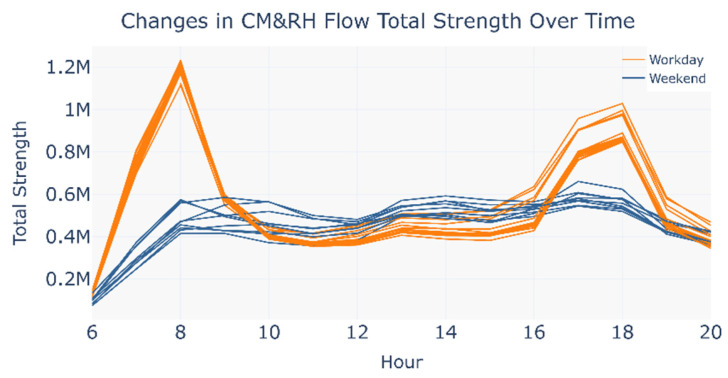
Changes in CM&RH flow total strength over time.

Due to the standardization of the feature matrices, the cosine similarity ignores vector magnitude differences, which may lead to the weakening of absolute flow differences between OD passenger flow matrices. To address this, the author supplemented the analysis with total strength variations in the CM&RH network (Figure 10).

The visualization results indicate that the variation trends of total strength did not exhibit significant divergence between workdays and weekends.

Based on the cosine similarity and variation in total passenger flow strength results, the following discussions are drawn.

(1) Monday to Thursday demonstrate commuting-oriented mode substitutability and self-organized intermodal networks.

The CM&RH network exhibits exceptionally high similarity (0.82) from Monday to Thursday, significantly exceeding the modal consistency of CM (0.70) and RH (0.68). This observation rules out the possibility that CM and RH passenger flows are independent, indicating that both networks share common underlying demand drivers and that these flow patterns occur extensively and systematically. Evidently, the passenger flows are primarily driven by rigid commuting demand. Such rigid demand motivates urban travelers to alternate between transport modes across different days, depending on real-time conditions such as extreme weather or time constraints.

**Figure 11:**
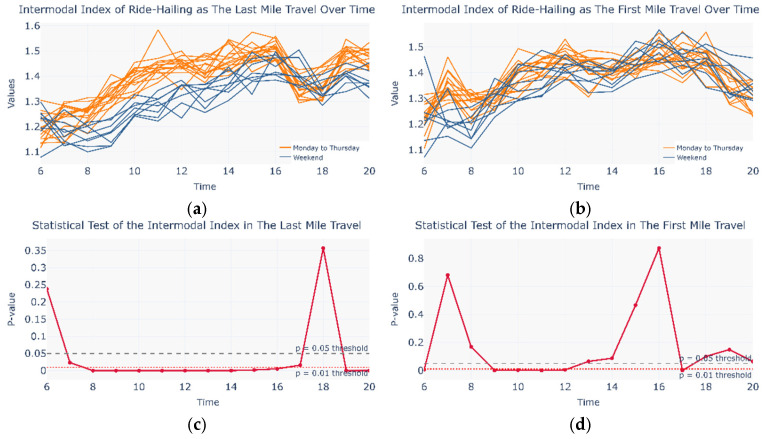
Intermodal coefficient and statistical test on Monday to Thursday and the weekend. (**a**) Intermodal coefficient in Monday to Thursday and Weekend in the Last Mile Travel; (**b**) Intermodal coefficient in Monday to Thursday and Weekend in the First Mile Travel; (**c**) Statistical Test in Monday to Thursday and Weekend in the Last Mile Travel; (**d**) Statistical Test in Monday to Thursday and Weekend in the First Mile Travel.

Flow patterns may occur not only across different travel days but also within a single day. During commuting periods, traffic pressure may prompt urban travelers to self-organize metro and ride-hailing services into an intermodal network. Since the cosine similarity matrix alone cannot effectively capture intra-day variations, this study defined an intermodal coefficient to examine temporal flow dynamics within a single day (see Equations (18) and (19)). Figure 11 compares the intermodal coefficient between Monday–Thursday and the weekend for both first-mile and last-mile scenarios, along with the corresponding Mann–Whitney U test results. Compared with the first-mile case, the last-mile scenario exhibits a wider range of time intervals with statistically significant differences, which are also more evident in the intermodal coefficient profile. The variation in the last-mile intermodal coefficient indicates that between 8:00 and 16:00, the weekday intermodal coefficient is significantly higher than that on weekends, which aligns well with actual commuting demand patterns. These findings suggest that during commuting periods, a portion of urban travelers spontaneously form a CM&RH intermodal network, in which ride-hailing services primarily fulfill the last-mile travel task.

(2) Divergence in Friday population flow patterns.

**Figure 12:**
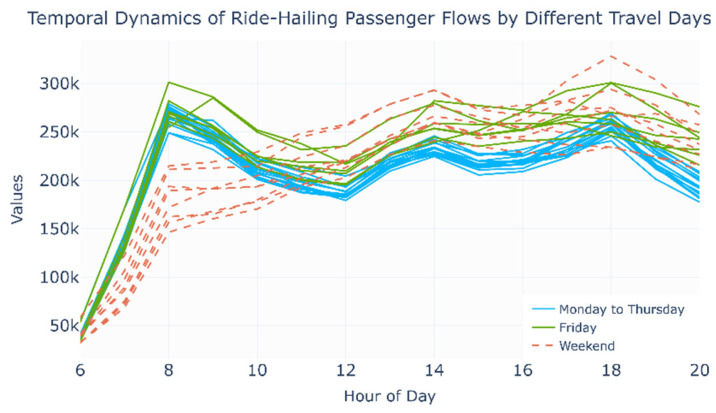
Temporal dynamics of ride-hailing passenger flows by different travel days.

On Fridays, the metro network maintains a similarity of 0.71—indicating that the core commuting cohort continues to rely on the metro network—whereas the ride-hailing network similarity drops sharply from 0.68 to 0.41. Figure 12 shows the hourly distribution of ride-hailing passenger volumes, where the flows on Fridays are higher than those from Monday to Thursday. This shift suggests that ride-hailing attracts a greater share of new users on Fridays, potentially because some commuters switch early to a “weekend mode,” using ride-hailing for flexible commuting or leisure trips. Overall, the mode substitutability between metro and ride-hailing weakens, the separation of commuting and leisure service scenarios emerges, and the overall consistency of the CM&RH network falls from 0.82 to 0.48.

(3) Leisure-oriented mode substitution and self-organized intermodal networks on weekends.

With commuting users absent, weekend travel patterns in both the metro and ride-hailing networks diversify; their similarities decline to 0.58 and 0.49, respectively, while the CM&RH network similarity remains at 0.72. This indicates that even non-rigid, leisure-oriented travel demands (e.g., shopping or social activities) can drive urban travelers to alternate modes across days.

**Figure 13:**
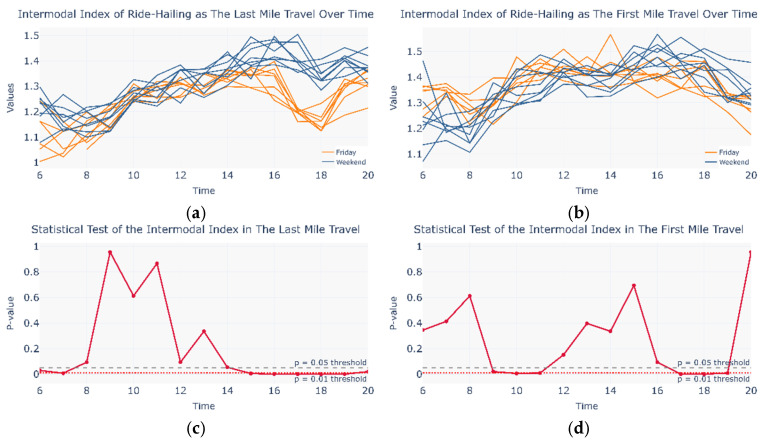
Intermodal coefficient and statistical test on Friday and the weekend. (**a**) Intermodal coefficient in Friday and Weekend in the Last Mile Travel; (**b**) Intermodal coefficient in Friday and Weekend in the First Mile Travel; (**c**) Statistical Test in Friday and Weekend in the Last Mile Travel; (**d**) Statistical Test in Friday and Weekend in the First Mile Travel.

To further investigate intra-day self-organized intermodal behavior, the author compares Fridays and weekend travel days—both lacking commuter characteristics. As shown in Figure 13, under leisure-oriented travel demand, the last-mile scenario exhibits a broader range of time intervals with statistically significant differences, which are also more pronounced in the intermodal coefficient profile. The variation of the last-mile intermodal coefficient indicates that after 15:00 on weekends, a portion of urban travelers spontaneously form a CM&RH intermodal network, in which ride-hailing services primarily fulfill the last-mile travel task. Furthermore, a transient ride-hailing–metro intermodal network may occur from 09:00 to 11:00 and from 17:00 to 19:00 on weekends, though its presence is not distinctly reflected in the inter-modal coefficient profile.

### 4.2. Sensitivity of the Intermodal Index to Network Mapping Methods

**Figure 14:**
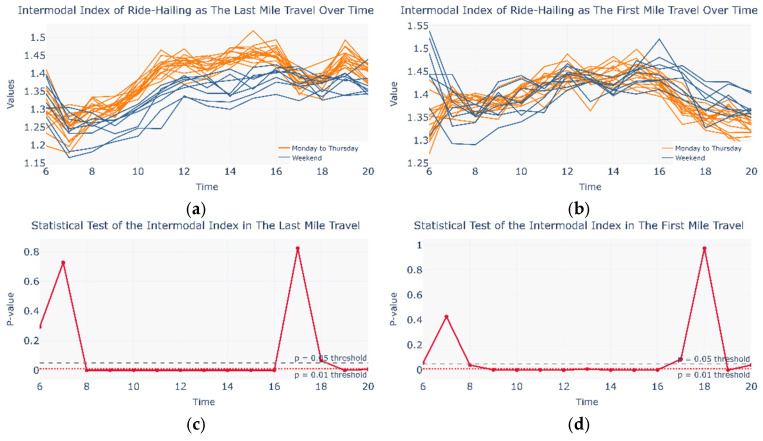
Comparison of intermodal coefficients and statistical tests under fully saturated mapping between Monday to Thursday and weekends. (**a**) Intermodal coefficient in Monday to Thursday and Weekend in the Last Mile Travel; (**b**) Intermodal coefficient in Monday to Thursday and Weekend in the First Mile Travel; (**c**) Statistical Test in Monday to Thursday and Weekend in the Last Mile Travel; (**d**) Statistical Test in Monday to Thursday and Weekend in the First Mile Travel.

**Figure 15:**
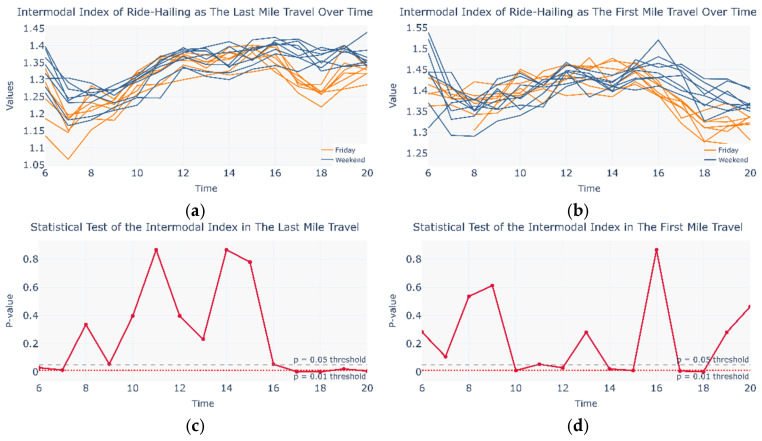
Comparison of intermodal coefficients and statistical tests under fully saturated mapping between Friday and the weekend. (**a**) Intermodal coefficient in Friday and Weekend in the Last Mile Travel; (**b**) Intermodal coefficient in Friday and Weekend in the First Mile Travel; (**c**) Statistical Test in Friday and Weekend in the Last Mile Travel; (**d**) Statistical Test in Friday and Weekend in the First Mile Travel.

In addition to the original mapping method, this study constructed a “saturated mapping” control group and calculated the distribution of the intermodal index under both mapping methods (see Figure 14 and Figure 15) to verify the sensitivity of the intermodal index. The results show that, regardless of the network mapping approach employed, the distribution of the intermodal index remains broadly consistent. This further confirms the widespread existence of self-organized intermodal networks and indicates that changing the mapping area has minimal impact on the core indicators.

### 4.3. Identify the Potential Intermodal Node

Clearly identifying intermodal nodes is a prerequisite for constructing intermodal transportation networks, particularly under resource-constrained conditions where prioritizing the importance of each intermodal node becomes critical [54,55,56]. Analysis of the intermodal index reveals that during periods when self-organized intermodal networks emerge, passenger flows within the mapped units exhibit high density. Given the difficulty of obtaining cross-network transfer passenger data, this study proposes utilizing the rich-club phenomenon to identify intermodal nodes. During periods of self-organized intermodal network formation, if both the metro and ride-hailing networks exhibit rich-club effects and their rich nodes show valid mapping relationships, it can be inferred that these rich nodes collectively serve, to a certain extent, as hubs for transfer flows.

Due to the independent operation of current transportation modes, rich-node distributions based solely on two real networks are insufficient to capture potential cross-network intermodal relationships. Therefore, the author introduces the concept of a potential intermodal network by constructing “RH-CM-RH” travel chains. A metro station is identified as a potential intermodal node only if it is recognized as a mutually mapped rich node in two real networks and simultaneously as a rich node in the potential intermodal network. Finally, the importance coefficients of potential intermodal nodes during commuting and leisure periods are determined, as shown in Equations (28) and (29).

#### 4.3.1. Positive Rich-Club Phenomenon and Secondary Rich Nodes

**Figure 16:**
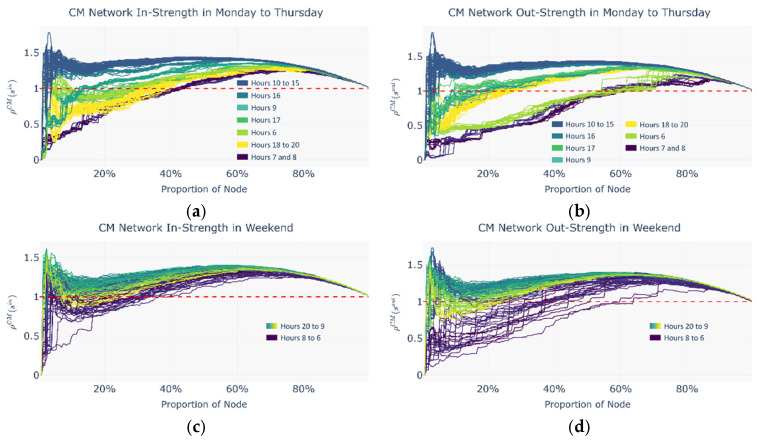
Distribution of normalized rich-club coefficients by node proportion (dynamic thresholds) during commute and leisure periods in the CM. (**a**) In strength of CM Monday to Thursday; (**b**) out strength of CM Monday to Thursday; (**c**) in strength of CM on the weekend; (**d**) out strength of CM on the weekend.

**Figure 17:**
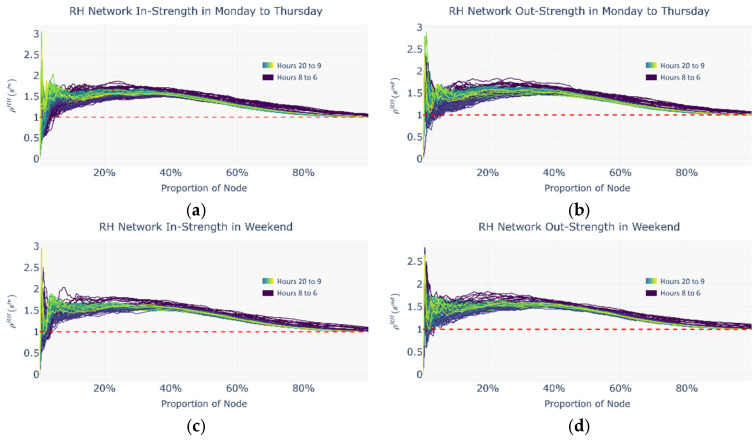
Distribution of normalized rich-club coefficients by node proportion (dynamic thresholds) during commute and leisure periods in the RH. (**a**) In strength of RH Monday to Thursday; (**b**) out strength of RH Monday to Thursday; (**c**) in strength of RH on the weekend; (**d**) out strength of RH on the weekend.

**Figure 18:**
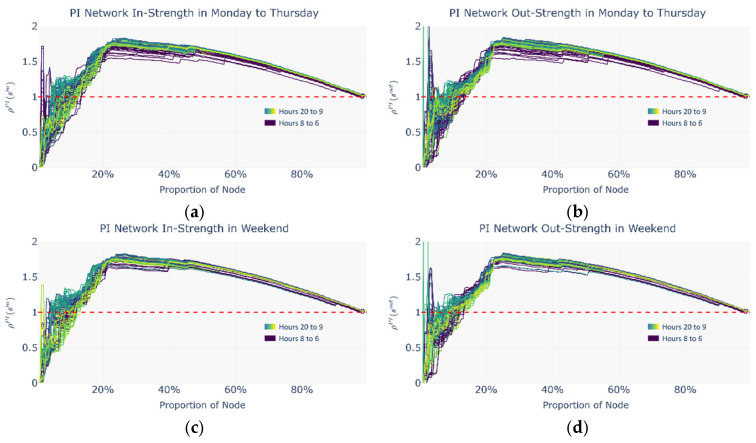
Distribution of normalized rich-club coefficients by node proportion (dynamic thresholds) during commute and leisure periods in the PI. (**a**) In strength of PI Monday to Thursday; (**b**) out strength of PI Monday to Thursday; (**c**) in strength of PI on the weekend; (**d**) out strength of PI on the weekend.

Based on the intermodal index analysis, the selected periods are classified into two categories: the commuting period, defined as Monday to Thursday from 7:00 to 18:00, and the leisure period, defined as weekends from 14:00 to 21:00. For consistency of visualization, all distribution figures display the complete 15 h data range. Figure 16, Figure 17 and Figure 18 present the distributions of the normalized rich-club coefficient *ρ*(*r*) with respect to the proportion of nodes under commuting and leisure periods and across the metro network, the ride-hailing network, and the potential intermodal network, separately for in strength and out strength. In these figures, the node proportion increases as the threshold decreases.

Overall, a significant positive rich-club phenomenon is observed across all periods, as indicated by the existence of intervals where the hourly normalized rich-club coefficient exceeds 1, implying that the connectivity strength among rich nodes surpasses the expected level in randomized networks. Moreover, each hourly normalized rich-club coefficient curve exhibits a characteristic bimodal pattern as follows:

(1) In the low node-proportion range, the curve rises sharply to the first local maximum and then rapidly falls to a local minimum;
(2) In the medium-to-high node-proportion range, the curve gradually rises again to a secondary peak before eventually declining towards 1.

This bimodal pattern likely reflects a hierarchical organization of the rich-club structure as follows:

(1) In the low node-proportion range, a core rich-club consisting of the richest nodes forms, characterized by highly dense internal connectivity;
(2) In the medium-to-high range, a secondary rich-club group emerges, incorporating both core and near-core nodes, revealing the network’s multi-scale hierarchical structure.

Since the core rich-club nodes account for a very small proportion (3–5%) and exhibit greater fluctuations in the normalized rich-club coefficient in the low node-proportion range, this study selects the more structurally stable secondary rich-club group as the basis for identifying potential intermodal nodes.

(1) Normalized rich-club coefficients in the CM

Figure 16 shows that the most prominent secondary rich-club groups in the CM cover 59.51% of all network nodes on average. In the commuting scenario, the hourly distribution of the normalized rich-club coefficient *ρ*(*r*) varies markedly. A clear core rich-club emerges only between 10:00 and 16:00, whereas no such concentration appears during the morning peak (07:00–08:00). In the leisure scenario, the hourly *ρ*(*r*) values are more tightly clustered, and from 09:00 onward, the CM exhibits a sustained hierarchical rich-club structure.

(2) Normalized rich-club coefficients in the RH

Figure 17 indicates that the most prominent secondary rich-club groups in the RH encompass 25.59% of nodes on average. Across both time periods, the hourly *ρ*(*r*) distributions are concentrated. Unlike the distribution in the CM, however, the hierarchical rich-club structure in the RH is unstable. The normalized coefficients for core rich nodes fluctuate sharply and irregularly as the threshold descends, suggesting that core rich nodes do not reliably reflect passenger flow characteristics.

(3) Normalized rich-club coefficients in the PI

Figure 18 reveals that the primary secondary rich-club groups in the PI cover 24.35% of nodes on average. Moreover, because the PI represents a potential—not actual—passenger flow network, its mobility characteristics bear limited real-world significance.

#### 4.3.2. Potential Intermodal Node and Importance Coefficient

Based on the distribution of the most prominent secondary rich-club groups in the three networks, the author uses the established node-mapping relations to identify a set of potential intermodal nodes and computes their importance coefficients under four scenarios—distinguishing travel purpose (commuting vs. leisure) and service mode (first-mile vs. last-mile)—as summarized in Table 5.

It can be seen that the number and distribution of potential intermodal nodes in the four scenarios are different: first-mile commuting requires the most nodes (22), leisure last-mile requires the fewest (12), and leisure first-mile and commuting last-mile require 20 and 18 nodes, respectively.

Several stations stand out. Chunxi Road station ranks highest (*IC* = 1) across all four scenarios; People’s Park station ranks second and has a higher *IC* during leisure than commuting; and Lijiatuo, Qianfeng Road, Erxianqiao, and Shengxian Lake stations appear only in the commuting scenarios, indicating no intermodal transfer value during leisure periods.

**Figure 19:**
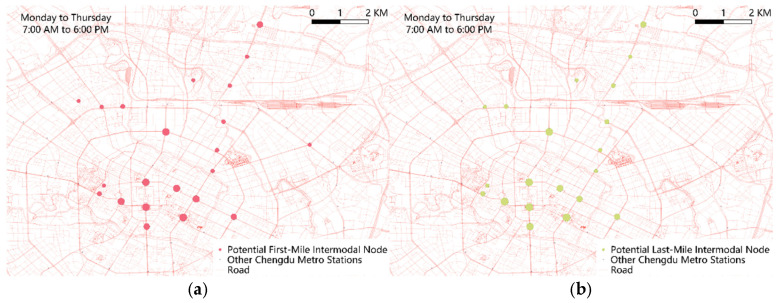

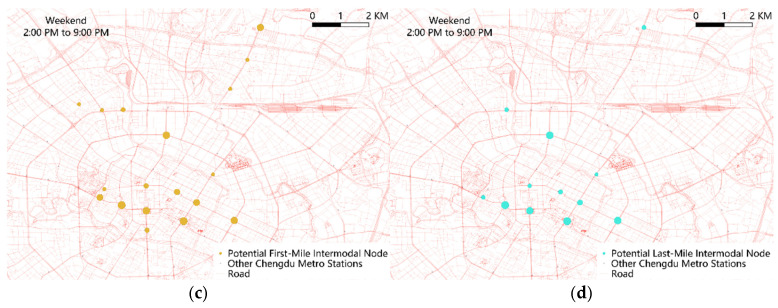
Potential intermodal nodes differentiated by trip purpose and first-/last-mile services. (**a**) Potential first-mile intermodal node of commute; (**b**) potential last-mile intermodal node of commute; (**c**) potential first-mile intermodal node of leisure; (**d**) potential last-mile intermodal node of leisure.

Figure 19 maps these potential intermodal nodes, with node size proportional to *IC*. The spatial distribution of potential intermodal nodes is heavily concentrated in Chengdu’s central area and radiates along an east–west axis, suggesting that north–south travel volumes exceed those east–west. A 2020 Chengdu study [57] reported that uneven regional development yields markedly higher southbound commuting flows than northbound. In the Dafeng subdistrict of northern Xindu District, residential density is 24,000 persons/km^2^ versus an employment density below 2500 persons/km^2^, whereas in the southern High-Tech South District, employment density reaches 15,000 persons/km^2^. Coupled with Chengdu’s urban layout—older, residential-oriented development in the north versus newer, commercial-industrial development in the south—this points to a pronounced north–south jobs–housing imbalance. Notably, most potential intermodal nodes outside the central core lie within Xindu District’s high-density residential zones.

### 4.4. Testing for Self-Organizing Phenomena

**Figure 20:**
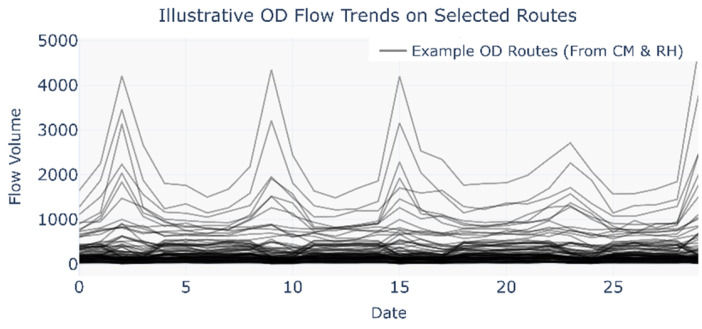
Illustration of daily OD flow trends on selected routes.

Due to the lack of corresponding passenger *ID* across the selected datasets, this study tests the self-organizing phenomenon by analyzing and comparing actual OD flow variations. As shown in Figure 20, the daytime OD flows of the sample routes from the CM and RH networks show certain regular patterns. Therefore, by comparing the correlation of flow variations on the same OD routes between the two networks, the self-organizing transfer phenomenon can be verified.

This study identifies two types of self-organizing phenomena: travel-mode substitution across days and within-day transfer networks. Based on these characteristics, key subway transfer nodes were determined. By selecting specific pairs of these nodes, travel-mode substitution can be analyzed through competing routes, while within-day transfer patterns can be examined via transfer routes.

**Figure 21:**
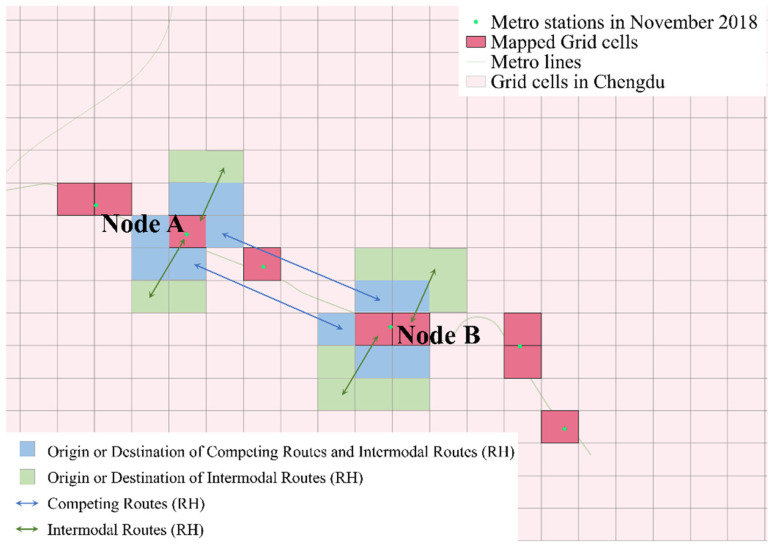
Schematic of the route for correlation analysis.

As illustrated in Figure 21, red blocks represent subway node areas; blue blocks indicate the origins and destinations of competing routes between nodes A and B, with blue bidirectional lines representing routes competing with the subway; and green blocks indicate the origins and destinations of transfer routes, with green bidirectional lines representing routes cooperating with the subway. In practice, a competing block may also serve as a transfer block, but a transfer block cannot be counted as competing. Additionally, blocks located too far from subway nodes are excluded from consideration as transfer-route blocks; in this study, the maximum distance is set at three intermediate blocks.

Two comparison groups were established in this study as follows:

(1) The top ten key transfer subway nodes, ranked by the node importance coefficient;
(2) Ten randomly selected non-key subway nodes.

For the key transfer node group, 90 unidirectional metro routes were constructed (including opposite-direction ODs), while the random node group yielded 72 unidirectional routes. The key node group generated 1814 unidirectional competing routes and 436 unidirectional transfer routes; the random node group generated 1490 competing routes and 364 transfer routes.

To enable correlation analysis, the flows of unidirectional competing routes between the same node pair were aggregated into a single route, and similarly, the unidirectional transfer-route flows associated with the same subway node were summed. In the correlation analysis of travel-mode substitution across days, competing-route flows were grouped by day; in the within-day transfer-network analysis, transfer-route flows were grouped by hour.

**Figure 22:**
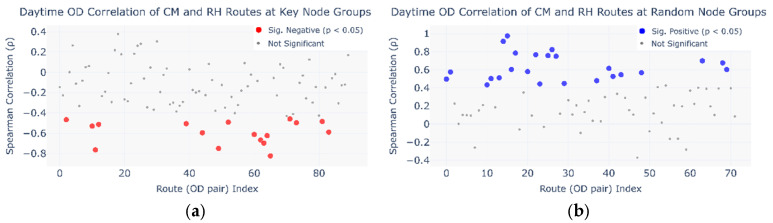

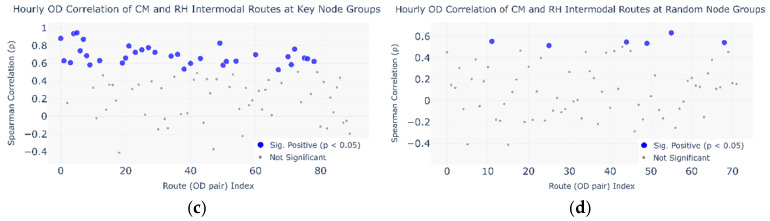
OD Correlation of CM and RH routes. (**a**) Daytime OD correlation of CM and RH routes at key node groups; (**b**) daytime OD correlation of CM and RH routes at random node groups; (**c**) hourly OD correlation of CM and RH intermodal routes at key node groups; (**d**) hourly OD correlation of CM and RH intermodal routes at random node groups.

The correlation analysis results are shown in Figure 22. By comparing Figure 22a,b, it is observed that a portion of the competing routes in the key transfer node group exhibit significant negative correlations between the CM and RH networks, whereas a larger proportion of competing routes in the random node group exhibit significant positive correlations. By comparing Figure 22c,d, a higher proportion of transfer routes in the key transfer node group shows significant positive correlations between the two networks.

The flow variations displayed in Figure 20 indicate a generally consistent trend of route-flow changes between the CM and RH networks, which aligns with the correlation results observed in Figure 22b–d.

The negative correlations observed on some competing routes in the key transfer node group are likely attributed to travel-mode substitution across days. Due to the high passenger volumes at these key nodes, riders may switch between the two transportation modes on different travel days under the influence of various factors, resulting in such negative correlations. In contrast, the positive correlations identified on some competing routes in the random node group suggest that mode substitution is less likely to occur on low-demand routes.

Similarly, the higher proportion of positively correlated transfer routes in the key transfer node group may stem from self-organizing transfer behavior, which may also exist to some extent in the random node group. Moreover, the correlation analysis results further confirm the validity of the proposed method for identifying key transfer nodes.

## 5. Conclusions

Emerging mobility modes enter urban transportation systems as fully independent layers yet interact with traditional public transit to produce a complex coopetition relationship. This phenomenon aligns with the Complex Adaptive Systems perspective: under specific external conditions (e.g., homogeneous demand), local agents make nonlinear, interdependent decisions that collectively give rise to ordered, coordinated structures beyond simple additive effects [57,58,59,60,61].

This study explicitly analyzes these emergent behaviors. When there exists a sizable and homogeneous demand cohort in both the metro and ride-hailing networks, city-scale collective behavior drives both modal substitution (competition relationship) and the formation of self-organized intermodal networks (cooperation relationship). Conversely, as demand diversifies and segments, substitutability weakens and the two networks’ service scenarios decouple. Under a commuting orientation, self-organized intermodal networks predominantly emerge between 08:00 and 16:00; under a leisure orientation, they form after 15:00. In either context, these networks consistently exhibit a ride-hailing last-mile feeder configuration.

These findings suggest that a key driver of intermodal network emergence is the presence of a sufficiently large, homogeneous demand cohort. Even without centralized, system-wide planning, such cohorts can induce multilayer networks to self-organize into orderly structures marked by concurrent cooperation and competition. Capitalizing on these coopetition characteristics, the author further identifies potential intermodal nodes whose spatial distribution aligns with the city’s actual population flow patterns. Moreover, a hierarchical rich-club structure—consisting of a small core group of high-strength nodes and a larger secondary cohort—was particularly pronounced in the metro network.

Overall, this study empirically demonstrates a spontaneous emergence mechanism driven by large-scale homogeneous demand under decentralized operation. It provides an empirical foundation for designing unified cross-modal scheduling strategies and lays the groundwork for future multilayer analyses of complete urban transportation systems, including the integration of bus networks, bike sharing, and other modes. Moreover, as the proportion of AI-controlled vehicles continues to increase in urban transportation networks, future research will further explore intermodal coordination patterns under hybrid human–AI mobility systems [62].

## Figures and Tables

**Table 1:** Example of Chengdu ride-hailing passenger flow data.

Data
[[104.0689, 30.66593, 1,541,143,701.0, 1], [104.0689, 30.66593, 1,541,143,704.0, 1], …]
[[104.06285, 30.69422, 1,541,200,108.0, 1], [104.06285, 30.69422, 1,541,200,111.0, 0], …]
[[104.07084, 30.67528, 1,541,138,954.0, 0], [104.07082, 30.67532, 1,541,138,957.0, 0], …]

**Table 2:** Brief information about Chengdu Metro in November 2018.

Operational Metro Line	Number of Stations	Number of Transfer Stations
Chengdu Metro Line 1	35	5
Chengdu Metro Line 2	32	5
Chengdu Metro Line 3	17	5
Chengdu Metro Line 4	30	5
Chengdu Metro Line 7	31	8
Chengdu Metro Line 10	6	1

**Table 3:** Example of Chengdu Metro passenger flow data. “EntS” and “ExiS” represent the entry station and the exit station.

Card ID	Card Type	EntS Name (ID)	Entry Time	ExiS Name (ID)	Exit Time
/	One-way	Zoo (323)	8 November 2018 17:04	Moziqiao (332)	8 November 2018 17:28
/	Tianfutong	ChunxiRoad (330)	8 November 2018 17:07	Hi-TechZone (133)	8 November 2018 17:27
/	Tianfutong	ChunxiRoad (330)	8 November 2018 17:05	Hi-TechZone (133)	8 November 2018 17:25

**Table 4:** Cosine similarity across three travel day types in three networks.

Cosine Similarity	Monday to Thursday	Friday	Weekend
Chengdu Metro	0.70	0.71	0.58
Ride-Hailing	0.68	0.41	0.49
CM&RH	0.82	0.48	0.72

**Table 5:** Four importance coefficients of potential intermodal nodes.

Station	Commute First Mile	Commute Last Mile	Leisure First Mile	Leisure Last Mile
Chunxi Road	1	1	1	1
Tianfu Square	0.93	0.99	0.89	0.86
Renmin North Road	0.92	0.96	0.91	0.93
Luomashi	0.88	0.91	0.32	0.14
Taisheng South Road	0.84	0.75	0.59	0.25
Chengdu Second People’s Hospital	0.82	0.63	0.75	0.46
People’s Park	0.81	0.99	1	1
Panda Avenue	0.66	0.59	0.82	0.21
Jinjiang Hotel	0.66	0.84	0.32	0
Yushuang Road	0.61	0.59	0.84	0.91
Tonghuimen	0.25	0.30	0.71	0.16
Jiulidi	0.24	0.17	0.25	0.16
Lijiatuo	0.16	0.26	0	0
Qianfeng Road	0.13	0.07	0	0
Zoo	0.11	0.04	0.05	0
Southwest Jiaotong University	0.10	0.02	0.05	0
Hongxing Bridge	0.10	0.03	0.04	0.02
Zhaojuesi South Road	0.10	0.19	0.05	0
Kuanzhaixiangzi Alley	0.04	0.09	0.05	0
Erxianqiao	0.02	0	0	0
Shengxian Lake	0.02	0.01	0	0
Huazhaobi	0.02	0	0.04	0
Other Chengdu Metro stations	0	0	0	0

## Data Availability

The authors do not have permission to share data.
